# Takayasu arteritis in a young child

**DOI:** 10.1007/s00247-026-06662-7

**Published:** 2026-05-21

**Authors:** Elazir Barbosa Mota Di Puglia, Luiz Antônio Rodrigues dos Santos, Gabriela Christine da Silva Freire, Eloá Nunez, Clarissa Canella, Carolina Ávila de Almeida

**Affiliations:** 1 Federal University of Rio de Janeiro, Rio de Janeiro, Brazil; 2 Hospital Jutta Batista - Rede D’or, Rio de Janeiro, Brazil

**Figure:**
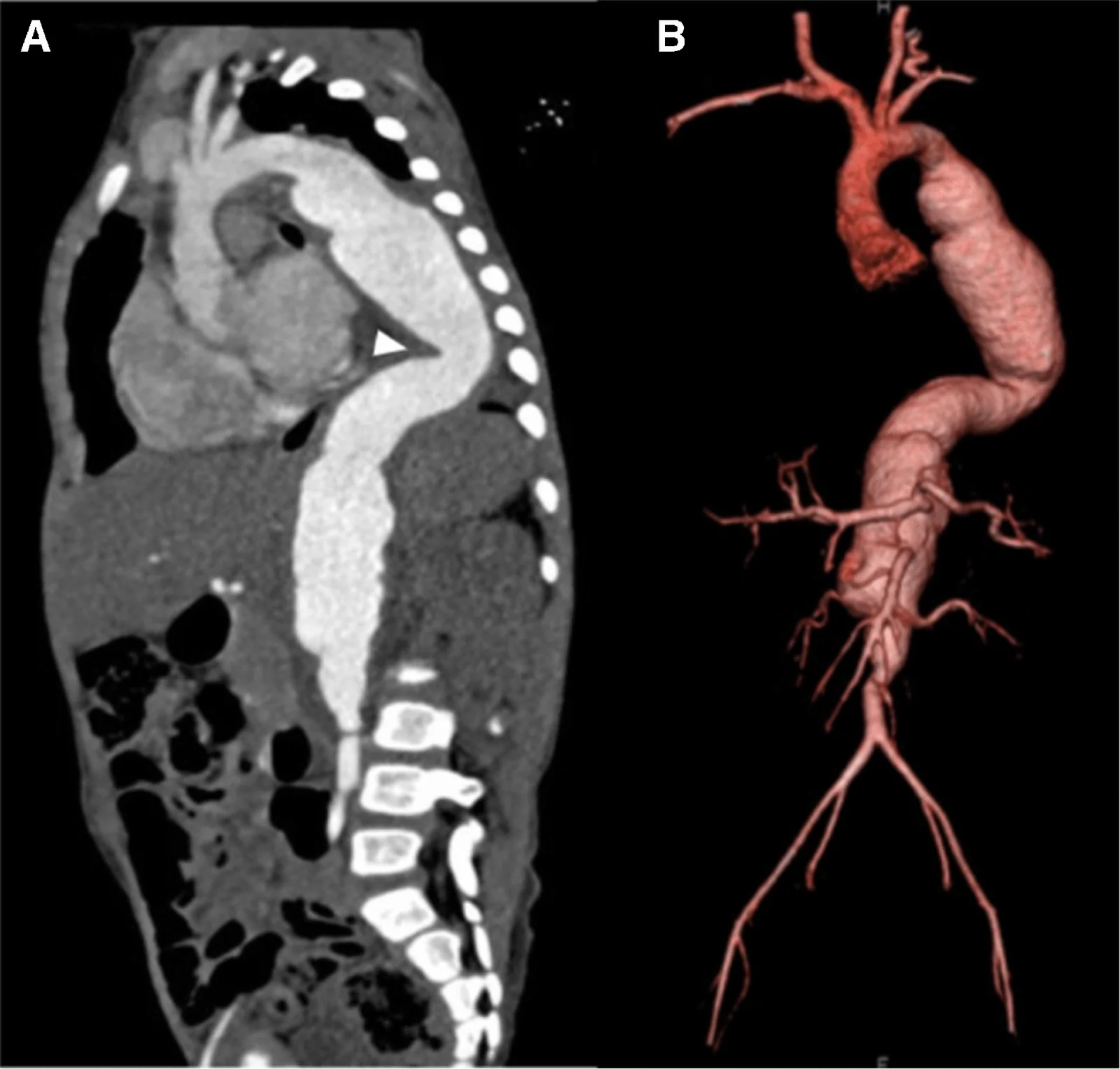

A 2-year-old girl with Takayasu arteritis. (**A**) Sagittal CT angiography reconstruction demonstrates a markedly dilated and tortuous thoracoabdominal aorta with focal kinking at the thoracoabdominal transition (arrowhead) (**B**) Three-dimensional volume-rendered CT angiography image shows irregular aneurysmal dilatation of the descending thoracic and abdominal aorta extending to the infrarenal segment.

## Data Availability

No datasets were generated or analysed during the current study.

